# The Regulatory Role of Oxygen Metabolism in Exercise-Induced Cardiomyocyte Regeneration

**DOI:** 10.3389/fcell.2021.664527

**Published:** 2021-04-15

**Authors:** Bing Bo, Shuangshuang Li, Ke Zhou, Jianshe Wei

**Affiliations:** ^1^Kinesiology Department, School of Physical Education, Henan University, Kaifeng, China; ^2^Sports Reform and Development Research Center, School of Physical Education, Henan University, Kaifeng, China; ^3^Institute for Brain Sciences Research, School of Life Sciences, Henan University, Kaifeng, China

**Keywords:** cardiomyocyte regeneration, exercise, oxygen metabolism, hypoxia, molecular pathway

## Abstract

During heart failure, the heart is unable to regenerate lost or damaged cardiomyocytes and is therefore unable to generate adequate cardiac output. Previous research has demonstrated that cardiac regeneration can be promoted by a hypoxia-related oxygen metabolic mechanism. Numerous studies have indicated that exercise plays a regulatory role in the activation of regeneration capacity in both healthy and injured adult cardiomyocytes. However, the role of oxygen metabolism in regulating exercise-induced cardiomyocyte regeneration is unclear. This review focuses on the alteration of the oxygen environment and metabolism in the myocardium induced by exercise, including the effects of mild hypoxia, changes in energy metabolism, enhanced elimination of reactive oxygen species, augmentation of antioxidative capacity, and regulation of the oxygen-related metabolic and molecular pathway in the heart. Deciphering the regulatory role of oxygen metabolism and related factors during and after exercise in cardiomyocyte regeneration will provide biological insight into endogenous cardiac repair mechanisms. Furthermore, this work provides strong evidence for exercise as a cost-effective intervention to improve cardiomyocyte regeneration and restore cardiac function in this patient population.

## Introduction

Heart failure (HF) is the primary cause of morbidity and mortality worldwide (Benjamin et al., 2019) and encompasses a variety of diseases impacting the heart and vasculature, leading to often fatal events, such as stroke, myocardial infarction (MI), and cardiac arrest (Benjamin et al., 2019; Pinckard et al., 2019). The adult mammalian heart is unable to regenerate lost or damaged cardiomyocytes at appropriate rates, and increasing evidence suggests that the mammalian heart is a postmitotic organ. Conversely, the neonatal heart has shown the ability to regenerate lost cardiomyocytes (Porrello et al., 2011). The adult heart has also demonstrated the ability to self-renew but at a much lower rate (Puente et al., 2014; Bergmann et al., 2015). The low rate of myocyte turnover that occurs in the adult heart is insufficient for the reconstitution of cardiac function in injured hearts (Nadal-Ginard, 2001; Bergmann et al., 2009). Cardiomyocyte regenerative capacity differs among species and life stages, closely related to the oxygen environment (Puente et al., 2014; Nakada et al., 2017). While oxygen-rich environments tend to induce cardiomyocytes to exit the cell cycle and lose regenerative ability after birth (Puente et al., 2014), hypoxia has been shown to activate cardiomyocyte mitosis through inhibited aerobic respiration and oxidative DNA damage in adult mice (Nakada et al., 2017). These studies suggest that alterations of the oxygen environment and metabolism play a vital role in cardiomyocyte regeneration.

For decades, the benefits of regular exercise for the therapy of heart disease have been widely recognized (Sanchis-Gomar et al., 2015; Verdoorn et al., 2017). The fundamental basis for the positive impacts of exercise on the heart is the increase in cardiac size and output (Pluim et al., 1998; Pluim et al., 2000). In elite athletes, the 10–20% greater cardiac dimension observed vs. healthy individuals has been attributed to cardiac hypertrophy (Maillet et al., 2013). In addition to our understanding that exercise causes cardiomyocyte hypertrophy, a growing body of research suggests that running or swimming may be able to activate the proliferation and regeneration capacity of cardiomyocytes (Waring et al., 2014; Vujic et al., 2018). Although the exact mechanism of exercise-induced cardiomyocyte regeneration is unclear, alteration of the oxygen environment and oxygen metabolism in the myocardium has been highlighted. This brief review will focus on exercise and oxidative metabolic control, concluding with evidence of how changes in oxygen metabolism may be involved in exercise-induced cardiomyocyte regeneration.

## Characteristics of Cardiomyocyte Regeneration

### Cardiomyocyte Regeneration Is Primarily Triggered by Cardiomyocyte Proliferation

Unlike lower vertebrates (e.g., zebrafish and newts) that maintain cardiac proliferation and differentiation ability for the entire lifespan (Poss et al., 2002; Porrello and Olson, 2014), the adult mammalian heart has been considered to have no capacity for proliferation. However, this traditional view has been challenged. It shows that the cardiomyocyte turnover rate is approximately 0.5–1% per year and almost 45% of cardiomyocytes are renewed throughout the human lifetime (Bergmann et al., 2009). Thus, it would appear that cardiomyocytes do have the ability to regenerate at low rates under typical physiological conditions. Previous studies have shown that endogenous cardiac precursor cells, including Sca-1^+^ cells (Wang et al., 2006; Uchida et al., 2013) and lsl-1^+^ cells (Moretti et al., 2006; Weinberger et al., 2012) play a limited role in cardiac regeneration during physiological and pathological conditions. In particular, Li Y. et al. (2018) used four different mouse transgenic models to demonstrate that non-cardiomyocytes do not contribute to new cardiomyocyte production during homeostasis or after injury in the adult heart. To date, the replacement rate of cardiomyocytes from preexisting cardiomyocytes is approximate 0.76% annually in younger adult rodent under physiological conditions. This rate decreases with age but increases by up to 4 times in post-MI regions (Senyo et al., 2013). Together, these studies suggest that the renewal of cardiomyocytes in newborn (Porrello et al., 2011; Li Y. et al., 2018), aging, and pathological conditions (Li Y. et al., 2018; Vagnozzi et al., 2018) is primarily derived from the proliferation of “preexisting” cardiomyocytes (Senyo et al., 2013). However, the reactivation and regulatory mechanisms of endogenous cardiomyocyte proliferation in adult hearts remain to be elucidated.

### Cardiomyocyte Regenerative Capacity Differs Among Species and Life Stages

**FIGURE 1:**
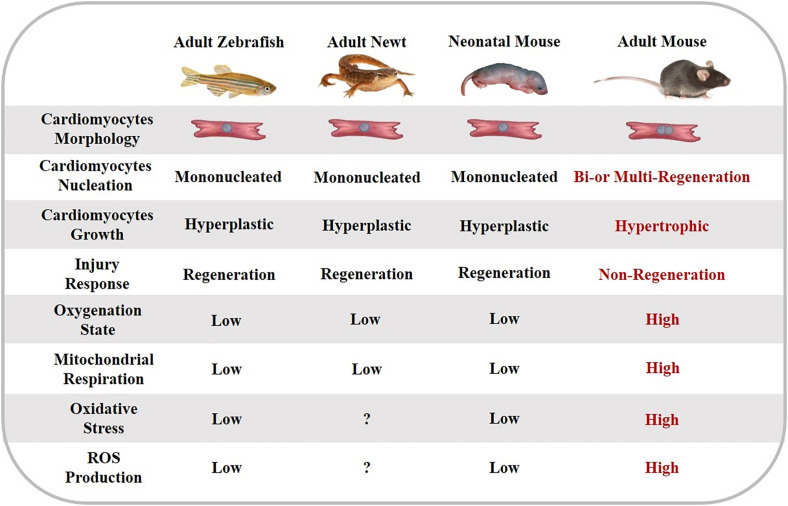
The characteristics of cardiomyocyte regeneration in different species and life stages.

During embryonic heart development, cardiac growth is primarily attributed to the division of existing cardiomyocytes (Galdos et al., 2017). After birth, the ability of cardiac regeneration differs among species and life stages. Adult non-mammalian animals (e.g., zebrafish, axolotls, and newts) are capable of regenerating the myocardium tissue throughout the lifespan (Poss et al., 2002; Jopling et al., 2010; Vagnozzi et al., 2018). For zebrafish, surgical amputation of up to 20% of the ventricle causes a remarkable increase of 5-Bromo-2′-deoxyuridine (BrdU)-labeled cycling myocytes (Poss et al., 2002). Similar regenerative phenomena have been reported in axolotls (Flink, 2002) and newts (Laube et al., 2006). After 60% ablation of zebrafish cardiomyocytes via genetic technology, all cardiomyocytes were replaced within 30 days (Chablais and Jazwinska, 2012). Moreover, the expansion of newborn cardiomyocytes can almost completely restore the structure and function of the ventricle. However, some studies also questioned whether this extent of regeneration is sufficient for more serious myocardial injuries or not (Figure 1) (Gonzalez-Rosa et al., 2017).

In contrast, the ability of cardiomyocyte regeneration is finite in adult mammalian hearts (Bergmann et al., 2009). However, the newborn mammalian heart preserves excellent regenerative ability during a short period following birth. Porrello et al. (2013) found that 1-day-old neonatal mice display sufficient regenerative capacity from preexisting cardiomyocytes after resection of ventricular apex (Porrello et al., 2011) and MI surgery. Accordingly, echocardiography in these studies has revealed that regenerated ventricular tissue is fully functional within 2 months (Porrello et al., 2011) and 3 weeks (Porrello et al., 2013), respectively. However, the mice’s heart has been shown to be incapable of regeneration 7 days after birth (Porrello et al., 2011, 2013), which is consistent with binucleation and arrest of the cardiomyocyte cell cycle (Soonpaa et al., 1996). Other studies have indicated that cardiomyogenic ability is limited in neonatal mice, which showed irreversible fibrosis, dilated cardiomyopathy (Andersen et al., 2016), and persistent scarring (Zebrowski et al., 2017) following apex resection. In neonatal porcine that undergo MI surgery, cardiomyocyte proliferation only lasts 2 days after birth, after which cardiomyocytes exit from the cell cycle, and contractile functions of the ventricle are damaged (Ye et al., 2018; Zhu et al., 2018). In a newborn child who suffers from severe MI due to coronary artery occlusion, clinicians have observed cardiac functional recovery within weeks after the initial ischemic insult. This phenomenon suggests that newborn human cardiomyocytes may preserve the intrinsic ability to replace injury cardiomyocyte and restore cardiac function entirely (Haubner et al., 2016). Moreover, cardiomyocyte proliferation is remarkable in newborn human hearts under pathological conditions (Farooqi et al., 2012; Nakagama et al., 2018).

Compared with adult mammalian cardiomyocytes, the heart of neonatal mammalians and low vertebrates share reparative capabilities through cardiomyocyte proliferation in the myocardium (Gomes et al., 2016). Therefore, whether postmitotic stimulation that drives cardiomyocyte regeneration in zebrafish and neonatal mammalians can induce similar responses in adult mammalian hearts, is a meaningful question (Foglia and Poss, 2016).

## Role of Oxygen Metabolic Mechanism in Cardiomyocyte Regeneration

### Oxygen Environment of Cardiomyocytes Differ Among Species and Life Stages

Among the numerous regulators of cardiomyocyte regeneration, the oxygen environment has received increased attention (Puente et al., 2014; Kimura et al., 2015; Nakada et al., 2017; Sakaguchi et al., 2020). In different species and life stages, oxygen supply and metabolism are distinct. Compared to the air, the oxygen capacitance of the aquatic environment where zebrafish live is only 1/30th that of the air, which can be considered a reduced level of oxygenation. This extremely hypoxic context might explain the prominent tolerance of zebrafish to hypoxia (Rees et al., 2001; Roesner et al., 2006). The typical PaO_2_ of oxygen-saturated water is 146 mmHg. Zebrafish can tolerate a PaO_2_ of 15 mmHg (10% oxygen-saturation) for 48 h and even 8 mmHg (5% air-saturation) under hypoxic pretreatment (Rees et al., 2001). Moreover, the zebrafish heart is a primitive two-chamber organ, with a single atrium and single ventricle, which leads to the mixing of arterial and venous blood. Similarly, the mammalian fetal circulation carries hypoxic blood with an arterial PaO_2_ of 25–35 mmHg, mainly due to a large amount of arteriovenous mixing in the fetal circulation. After the newborn’s first breath, the transition from embryonic to postnatal circulation significantly changes the oxygenation of cardiomyocytes. Meanwhile, the mammalian heart is characterized by four chambers, with an arteriovenous shunt in the neonatal heart. Thus, arterial PaO_2_ immediately increases from 30 mmHg (Lawrence et al., 2008) to 100 mmHg (Webster and Abela, 2007). Compared to the relatively small regenerative capacity in the adult mammalian heart, the prominent cardiomyogenic capacity of zebrafish is very likely due to the “oxygen poor” environment. Therefore, the oxygen environment of cardiomyocytes plays a critical role in the maintenance and regulation of cardiomyocyte regeneration.

### Increased Oxygen Supply After Birth Induces a Metabolic Switch and Cessation of Cardiomyocyte Proliferation

During the embryonic period, anaerobic glycolysis is the essential energy supply for the hypoxic heart. Soon after birth, with enhanced oxygen capacitance (20% oxygen in the air), the mammalian heart experiences a rapid shift in energy utilization. In the early newborn period, glycolysis and lactate oxidation are still the central metabolic process that supply energy. Simultaneously, fatty acid β-oxidation provides less than 15% of the heart’s total adenosine triphosphate (ATP) requirements. The small amount of energy supplied by fatty acid β-oxidation is partly due to an inhibition of mitochondrial fatty acid uptake. However, within a few days following birth, a sharp increase in fatty acid β-oxidation coincides with a decrease in glycolytic rates. By 7 days postpartum, glycolysis is further reduced, and provides less than 10% of total ATP produced by cardiomyocyte metabolism, whereas fatty acid β-oxidation increase progressively to produce 60–80% of ATP in the adult heart under standard physiological contexts (Lopaschuk et al., 1992, 2010). A recent study indicates that inhibition of fatty-acid utilization improves cardiomyocyte proliferation through DNA damage reduction and DNA-damage response (DDR) pathway in the postnatal heart (Cardoso et al., 2020). Proliferator-activated receptor α (PPARα) regulates several genes that control mitochondrial import and oxidation of fatty acid in heat (Djouadi et al., 1999). Further study found that pharmacologic and genetic activation of PPARα-mediated fatty acid β-oxidation promoted hypertrophic cardiomyocyte growth and maturation, which induced cytokinesis failure and cell cycle exit. The etomoxir (ETO)-mediates inhibition of myocardium fatty acid β-oxidation metabolism enhanced glycolysis and maintained cardiac proliferation in newborn mouse hearts at postnatal days 5 and 7 (Cao et al., 2019). Another study detected enzyme activity related to glycolytic and mitochondrial metabolism in the early postpartum period. For example, enzymes of Krebs cycle and fatty acid oxidation increase within 7 days of birth, with a simultaneous reduction of anaerobic glycolysis (Puente et al., 2014). These observations suggest that oxygen-dependent mitochondrial oxidative phosphorylation is the primary energy supply in adult cardiomyocytes. Interestingly, the mammalian heart’s adaptability to high-concentration oxygen supply is synchronized with the stagnation of cardiomyocyte proliferation. However, the relationship between time window of metabolic shifts and cardiomyocyte regeneration in normal developmental and physiological settings remain unanswered.

The shift from anaerobic to aerobic metabolism is closely related to mitochondrial expansion in the myocardium, increase in reactive oxygen species (ROS) production, cardiomyocyte terminal differentiation, and cell cycle exit (Carley et al., 2014). Through comparison of the mitochondrial characteristics of zebrafish and newborn mouse hearts, it can be found that the mitochondrial expansion and cristae formation in mouse myocardial mitochondria is consistent with the time window in which cardiomyocyte regenerative ability is lost (Puente et al., 2014). Meanwhile, oxidative metabolism generates ROS through the mitochondria respiratory chain. ROS are produced by the secretion of the superoxide ion (O_2_^–^) due to electron leakage. This oxide can quickly transform into H_2_O_2_ and then into OH free radicals. A low concentration of ROS is harmless to cells and can be scavenged by antioxidants or the endogenous antioxidant pathway (Liaudet et al., 2009). However, the accumulation of ROS generates oxidative stress (Sarangarajan et al., 2017), inducing detrimental effects, such as oxidative nuclear DNA damage, proliferation, or inactivation of differentiation signaling pathways (Puente et al., 2014). It has been confirmed that some antioxidants (Pitx2 and TT-10) are able to enhance neonatal cardiomyocyte proliferation by activating the YAP signaling pathway (Puente et al., 2014; Tao et al., 2016; Hara et al., 2018). Previous studies have indicated that the process of proliferation in cardiomyocytes is associated with oxygen and aerobic respiration-mediated oxidative DNA damage (Puente et al., 2014; Kimura et al., 2015; Liu et al., 2015; Bon-Mathier et al., 2019). The primary source of oxidative stress in postnatal cardiomyocytes is mitochondrial-derived ROS (Adam-Vizi and Chinopoulos, 2006). The shift from the hypoxic uterine environment to the postpartum environment causes a mitochondrial ROS-induced oxidative DNA damage response (DDR), which results in the arrest of the cardiomyocyte cell cycle. Puente et al. (2014) also indicated that activation of ATM kinase in response to DNA damage, in turn, activates Wee1 kinase, a repressor of cyclin-dependent kinase 1 (CDK1)-dependent G2-M transition. Wee1 did not immediately express in the nuclei of cardiomyocytes after birth, but was strongly upregulated at postnatal days 7 and 14 (Puente et al., 2014). Therefore, inhibition of Wee1 could induce greater activity of endogenous CDK1 and cyclin B1 (CCNB) complex to facilitate the G2/M phase and cardiomyocyte mitosis (Bicknell et al., 2004; Harvey et al., 2005; Mohamed et al., 2018). Moreover, the elimination of ROS can downregulate Wee1 expression to extend the postnatal cardiomyocyte proliferation window (Puente et al., 2014). Low concentrations of ROS may be able to regulate the protective pathways of ischemic preconditions (Zhou et al., 2018). Furthermore, cardiomyocyte adaptation to progressively higher oxygen supply after birth coincides with proliferation stagnation in the adult heart. The main reason for this phenomenon is oxygen-dependent mitochondrial metabolism (Nakada et al., 2017) and ROS production upregulation (Bon-Mathier et al., 2019).

### Hypoxia and Cardiac Regeneration

The state of decreased oxygen availability is termed hypoxia and affects energy metabolism and contractile function of the myocardium (Essop, 2007; Cole et al., 2016). Chronic hypoxia can reduce mitochondrial fatty acid uptake and oxidation (Cole et al., 2016; Mansor et al., 2016), accompanied by decreased activity of fatty acid oxidation enzymes (Daneshrad et al., 2000; Kennedy et al., 2001; Heather et al., 2012), while glycose uptake (Hurford et al., 1990) and the activities glycolytic enzymes (Waskova-Arnostova et al., 2014; Cole et al., 2016; Mansor et al., 2016) increase after exposure to chronic hypoxia in the heart. Similarly, hypoxia reduces the mitochondrial respiration of fatty acid and pyruvate substrates, enzymatic activities of electron transport chain complexes I, II, and IV in subsarcolemmal mitochondria, and ROS generation (Heather et al., 2012). Metabolic alteration of the heart is primarily due to the shift from oxygen-fastidious fuels to oxygen-efficient substrates. One study group that exposed adult mice to a systemic gradual hypoxic environment (decreasing progressively in oxygen concentration by 1% per day from 20.9% ambient oxygen to 7% for 2 weeks) found that mitochondrial metabolism, ROS production, and oxidative DNA damage were reduced, all of which may be able to promote cell cycle reentry in differentiated cardiomyocytes at baseline or after injury (Nakada et al., 2017). A hypoxic environment may protect cardiomyocytes from ROS upregulation-induced oxidative DNA damage (Kikuchi et al., 2010). Additionally, hypoxia-induced metabolic reprogramming, cell cycle reentry, and regeneration in adult cardiomyocytes have been confirmed. Hypoxia not only promotes cardiomyocyte proliferation at the cellular level, but also plays an important role in heart regeneration. For example, exposure to hypoxia (7% inspired oxygen concentration) for 7 days could remarkably increase the BrdU-positive cardiomyocyte and capillary collaterals, which provides supports for the improvement of left ventricular systolic function following myocardial infarction in mice heart (Nakada et al., 2017).

During the adaptation of cells to hypoxia, hypoxia-inducible factors (HIFs) are the most critical regulatory factors. HIFs are a family of obligate heterodimers composed of unstable α-subunits (HIF-α) (Wang et al., 1995; Kallio et al., 1997). Three HIF-α isoforms exist in humans: hypoxia-inducible factor 1 alpha (HIF-1α), HIF-2α, and HIF-3α (Hu et al., 2003). HIF-1α protein is stable under hypoxia, leading to transcriptional activation of multiple target genes involved in glycolytic, fatty acid, and mitochondrial metabolisms, as well as cell cycle regulators (Kaelin, 2005; Prabhakar and Semenza, 2015). HIF-1 participates in hypoxia-induced inhibition of fatty acid metabolism by reducing the DNA binding activity of PPARα in rat cardiomyocytes (Belanger et al., 2007).

Additionally, the HIF-1α protein is necessary for cardiomyocyte regeneration in injured hearts of zebrafish (Jopling et al., 2012) and hypoxic cardiomyocytes of fetuses (Dunwoodie, 2009) by regulating the cell cycle and cellular metabolism (Breckenridge et al., 2013; Guimaraes-Camboa et al., 2015; Hubbi and Semenza, 2015; Menendez-Montes et al., 2016). In HIF-1α overexpressing transgenic mice, Kimura et al. (2015) used fate mapping to identify a few cardiomyocytes that preserved embryonic characteristics, including mononucleates, smaller cell size, lower levels of oxidative DNA damage, and contribution to cardiomyocyte regeneration. RNA sequencing analysis of isolated hypoxic proliferative cardiomyocytes indicated that an increase of HIF-1α and a decrease of prolyl hydroxylases protected hypoxic proliferative cardiomyocytes from oxidative stress (Kimura et al., 2015). In MI rats, the upregulation of HIF-1α protein and HIF-1 targets in mRNA have been detected within the first week after infarction (Hu et al., 2003). In pathological hearts, the ischemic tissue no longer receives oxygen or nutrients due to MI (Russell et al., 2004; Thygesen et al., 2018), resulting in the generation of free radicals and mitochondrial dysfunction by decreased utilization of fatty acid and increased employment of glycolysis and glucose oxidation (Sack et al., 1996; Davila-Roman et al., 2002). Therefore, ischemia inevitably induces local hypoxia in the affected tissue (Sousa Fialho et al., 2019).

These studies suggest that modulating oxygen levels or myocardial metabolism could be a novel method to induce new cardiomyocyte formation. This increase in cardiomyocyte renewal is likely due to decreased mitochondrial respiration, mitochondrial ROS production, and oxidative DNA damage under a systemic hypoxic microenvironment, thereby promoting cell cycle reentry. However, the risks associated with a hypoxic state of tissue need to be considered when transferring basic research results from small creatures to large animal models or human subjects.

## Possible Changes of Exercise-Induced Physiological Effects on Heart

### Classification of Exercise

During exercise, the energetic source of skeletal muscle is mainly from carbohydrates, fatty acids and amino acids. Based on differences in mechanical action and energy metabolism of muscles, exercise is classified into dynamic and static exercise (Levine et al., 2015). Dynamic and static exercises differ in their mode, intensity, frequency, and volume (Patel et al., 2017).

Dynamic exercise mainly utilizes large skeletal muscle groups that can be continuously maintained and have rhythmic physical activity (Wahid et al., 2016). Moderate to high-intensity dynamic exercise is performed aerobically. Aerobic capacity is the ability of cardiopulmonary system supplying oxygen to tissue and skeletal muscle utilizing oxygen (Thompson et al., 2013). Cardiac output can maintain exercise for approximately 20 min during dynamic exercise and starts to decline thereafter due, at least in part, to cardiac drift (Coyle and Gonzalez-Alonso, 2001). With the extension of exercise time and the accumulation of cardiopulmonary system fatigue, insufficient oxygen supply cannot meet tissue demands for oxygen, causing relative hypoxia of tissues (such as muscles and myocardial tissues) (Michailidis et al., 2007; Powers and Jackson, 2008; Cimen et al., 2017). Whether this hypoxic environment can initiate the cardiomyocyte regeneration signaling pathway through the oxygen metabolic mechanism remains unclear.

Static exercise is a kind of strenuous physical activity with a short time. It is performed anaerobically that is maintained by energy in the contracted skeletal muscles and does not use oxygen to participate in energy production (Thompson et al., 2013). In the absence of oxygen, our cells produce ATP through the phosphate and glycolysis pathways. This method produces much less ATP than the aerobic oxidation pathway and causes accumulation of lactic acid. Static exercise is generally considered to consist of fast-twitch muscles and includes sprinting, powerlifting, and high-intensity interval training (HIIT). Static exercise leads to a continuous increase in lactic acid, and a turning point called the lactate threshold (LT). The definition of the LT is the point where the metabolic energy supply mode shifts from aerobic metabolism to anaerobic metabolism, leading to a continued increase in lactic acid and metabolic acidosis (Wasserman, 1986; Floria and Mareev, 1993).

Different types of exercise can induce a variety of different types of oxygen supplementation and consumption in cardiomyocytes. However, the relationship between exercise program and oxygen metabolism (e.g., intensity, frequency, and volume) needs to be further evaluated according to the physiological and pathological conditions of the individual (Merghani et al., 2016).

### Potential Detrimental Effects of Exercise on the Heart

Although regular and appropriate exercise has been shown to affect cardiovascular function positively, strenuous acute exercise or chronic excessive exercise training resulting in excessive cardiac stimulation may be harmful. Animal and human studies have confirmed that excessive acute and chronic exercise can increase cardiovascular pressure, leading to an alteration in the pathological structure of main arteries and remodeling of myocardial electrophysiology. Simultaneously, the atrium and right ventricle (RV) can experience transient acute volume overload, which leads to RV hypokinesia and diastolic dysfunction due to a sharp increase in returning blood volume caused by exercise (Parry-Williams and Sharma, 2020). Moreover, inflammation and fibrosis of the atria, ventricular septum, and RV of repeated overstimulation and injury induced by excessive exercise might be the essential reason of atrial and ventricular arrhythmias (O’Keefe et al., 2012). Therefore, the effect of exercise on heart structure and function is directly related to the type, intensity, and duration of the exercise program.

## Exercise-Induced Oxygen Metabolism Changes in Cardiomyocyte Regeneration

**FIGURE 2:**
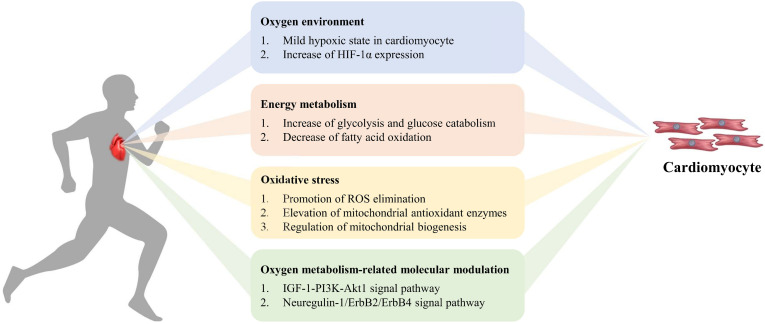
Exercise-induced oxygen metabolism alteration in cardiomyocyte regeneration. During exercise, mild hypoxia and increase of HIF-1α expression appear in cardiomyocytes. The increase of glycolysis and decrease of fatty acid oxidation influence energy metabolism, to promote ROS elimination, elevate mitochondrial antioxidant enzymes activity, enhance mitochondrial biogenesis, and to improve myocardial antioxidant capacity. Simultaneously oxygen metabolism-related molecular modulation activates cardiomyocyte proliferation via signal pathway.

Regular exercise can result in changes in the cardiovascular and respiratory systems, through an increase in the provision of oxygen to working skeletal muscles and other tissues. Cardiac growth induced by exercise could improve myocardial contractility, arterial blood flow, and reduce myocardial ischemia in the mammalian heart (Duncker and Bache, 2008; Lavie et al., 2015; Platt et al., 2015; Vujic et al., 2018). However, the complex molecular and cellular mechanisms of exercise-induced cardiomyocyte renewal are ambiguous. In the following section, we will focus on the oxidative mechanisms involved in cardiomyocyte regeneration during and after exercise (Figure 2). Whether there is a dose-response effect due to exercise duration and intensity is debatable (Patel et al., 2017).

### Exercise-Induced Oxygen Environment Variation in Cardiomyocytes

At rest, the total volume of ejected blood is ∼7,200 L per day, which requires the heart to pump ∼100,000 times and consume ∼10–20% of the body’s oxygen. To maintain this amount of work, the heart depends on high capillary density (∼3,500 capillaries per mm^2^), continuous blood flow (∼250 mL min^–1^), and fatty acid oxidation (supplying 40–70% of cardiac ATP) (Laughlin and Tomanek, 1987; Duncker and Bache, 2008). As the most essential physiological stimulus, exercise increases myocardial contractility and oxygen consumption 3–10 × that of resting rates (Lopaschuk et al., 2010; Olver et al., 2015). To meet the elevated oxygen demand during exercise, myocardial blood perfusion increases from 0.8 mL/g⋅min at rest to 3.2 mL/g⋅min at peak exercise, improving overall oxygen supply (Gielen and Hambrecht, 2001; Duncker and Bache, 2008). Compared to untrained individuals, exercise leads to an approximate 20–100% increase in left ventricle mass (Kozakova et al., 2000; Zandrino et al., 2000). As exercise-induced cardiac mass increases, matched angiogenesis should occur so that capillary density is maintained in an effort to satisfy the new cardiomyocyte oxygen and nutrient demands. The capillary-myocyte ratio is used to determine capillary density in the heart. It has been demonstrated that the capillary density in dogs (Wyatt and Mitchell, 1978; Laughlin and Tomanek, 1987) and swine (Breisch et al., 1986) does not show an upward trend after exercise. In swine, although the division of coronary artery endothelial cells and the sprouting of capillaries increases at 1, 3, and 8 weeks, this alteration disappears after long-term exercise intervention (i.e., 16 weeks) and displays no difference from a sedentary group (White et al., 1998). This could mean that ventricle and capillary growth occur at different rates. Nevertheless, this mismatch between cardiomyocyte growth and capillary density increase may induce a mild hypoxic state in myocytes during and after exercise.

As the mediator of physiological and pathophysiological responses to hypoxia, HIF-1α might be an essential indicator of the exercise-induced oxygen environment in the myocardium (Table 1). After 8 weeks of treadmill running, HIF-1α mRNA expression increases in adult mice. Moreover, the upregulation of HIF-1α also increases glucose transporter 1 (GLUT1) and lactate dehydrogenase A (LDHA) expression, which are related to significant metabolic adjustment in the myocardium (Vujic et al., 2018). A recent study indicated that mild-intensity exercise has a more significant effect on the increase of HIF-1α upregulation than moderate- and high-intensity exercise (Bellafiore et al., 2019). Both dynamic and static exercise can increase the expression of HIF-1α in cardiac tissue. Static exercise has a more substantial effect on upregulating HIF-1α expression, as glycolysis is its primary energy supply (Flora et al., 2012). Under pathological conditions, 8 weeks of treadmill running could significantly increase the expression of HIF-1α and vascular endothelial growth factor (VEGF) in transverse aortic constriction mice (Tian et al., 2020). Additionally, 4 weeks of HIIT consisting of running exercise also increased HIF-1α content in the cardiac tissue of hypertensive rats (Holloway et al., 2015). However, other studies have suggested that HIF-1α content decreases in response to exercise training (Pinho et al., 2012), or remains unchanged (Sylviana et al., 2018). These discrepancies might be due to model-specific factors, such as the program of exercise and animal strain. Taken together, these findings suggest that the relationship between HIF-1α and cardiomyocyte regeneration within the exercise context is ambiguous. Further studies are needed to probe the specific effects of HIF-1α under exercise-induced alteration of the oxygen environment.

**TABLE 1:** Factors related to oxygen mechanisms involved in exercise-induced cardiomyocyte regeneration.

Classification	Factor	Species	Exercise model	Duration	Observation	References
Oxygen environment	HIF-1α	Mice	Running exercise: voluntarily running.	8 weeks 5 days/week	HIF-1α↑; New cardiomyocytes in adult mice ↑	Vujic et al., 2018
	HIF-1α	Mice	Running exercise: rotating treadmill, training intensity performed by 15, 30, and 45 days corresponded to a mild, moderate, and high intensity, respectively.	15, 30, and 45 days 5 days/week	HIF-1α↑	Bellafiore et al., 2019
	HIF-1α	Mice	Running exercise: ramp protocol increased from 11 m/min for 30 min/day to 13 m/min for 60 min/day.	8 weeks 5 days/week	HIF-1α and VEGF ↑	Tian et al., 2020
	HIF-1α	Rat	Running exercise: 20 m/min at a 10° incline, the session lasted 8 min in week1, 15 min in week 2, and 23 min in weeks 3 and 4.	4 weeks 5 days/week	HIF-1α↑	Holloway et al., 2015
	HIF-1α	Rat	Running exercise: aerobic exercise protocol is 20 m/min for 30 min/day, anaerobic exercise protocol is 35 m/min for 20 min/day.	1, 3, 7, and 10 days	HIF-1α and VEGF ↑;	Flora et al., 2012
Energy metabolism	Glycolysis	Mice	Swimming exercise: ramp protocol increased from 10 min/day to two 90-min sessions, twice/day, the sessions were separated by at least a 4-h interval	4 weeks 5 days/week	Glycolysis rate ↑	Riehle et al., 2014
	Glycolysis	Mice	Running exercise: 22.3 m/min at a 10° incline, the session lasted 40 min in week 1, 50 min in week 2, and 60 min in weeks 3 and 4.	4 weeks 5 days/week	Glycolysis decreased during exercise, but steady-state rates of glycolysis increased in the early and full recovery period after exercise.	Gibb et al., 2017
	Fatty acid oxidation	Mice	Running exercise: consisted of 10 bouts of 4 min high intensity training, corresponding to 85–90% VO_2__*max*_, interspersed by 2 min active rest, speed gradually from 16 to 26 m/min	10 weeks 5 days/week	Fatty acid oxidation ↓	Hafstad et al., 2011
Mitochondrial biogenesis	ROS	Rat	Running exercise: 25 m/min at a 6° incline, the session lasted 40 min in week 1, 50 min in week 2, and 60 min in weeks 3 and 4.	16 weeks 5 days/week	ROS production ↓	Starnes et al., 2007
	ROS	Rat	Running exercise: endurance training ramp protocol increased from 10 m/min for 30 min/day up to 15 m/min for 60 min/day	5 weeks 6 days/week	ROS production ↓; ROS elimination ↑	Bo et al., 2008
	AMPK/PGC-1α	Mice	Running exercise: endurance training 1.0 km/h for 60 min/day	16 weeks 5 days/week	AMPK/PGC-1α signal transduction ↑	Wang et al., 2020
	Sirt1/PGC-1α	Rat	Running exercise: endurance training ramp protocol increased from 4.2 m/min up to 12 m/min for 30 min/day	36 weeks 4-5 days/week	Sirt1 and PGC-1α↑	Bayod et al., 2012
	Sirt1/PGC-1α/PI3K	Rat	Running exercise: ramp protocol increased from 10 m/min for 30 min/day increased to 16 m/min and 60 min/day	4 weeks 7 days/week	Sirt 1/PGC-1α/PI3K signaling transduction ↑; oxidative stress ↓	Jia et al., 2019
	PGC-1α	Mice	Swimming exercise: moderate intensity lasted 30 min/day.	4 weeks 5 days/week	PGC-1α↑	Sylviana et al., 2018

*AMPK, adenosine monophosphate-activated protein kinas; HIF-1α, hypoxia-inducible factor 1α; PGC-1α, peroxisome proliferator-activated receptor-γ coactivator 1α; PI3K, phosphoinositide 3-kinase; ROS, reactive oxygen species; Sirt1, NAD-dependent deacetylase sirtuin1; VEGF, vascular endothelial growth factor; VO_2max_, maximum volume of oxygen consumption. ↑, upregulation; ↓, downregulation.*

### Exercise-Induced Cardiac Energy Metabolism Changes in Cardiomyocytes

During exercise, more factors regulate cardiac energy metabolism. First, the increased local and circulating catecholamines (i.e., epinephrine and norepinephrine) elevate heart rate and myocardial contractility. Meanwhile, alterations in cardiac workload (specifically preload and afterload) augment changes to substrate metabolism in the heart, increasing energy demand (Taegtmeyer et al., 2016a; Gibb and Hill, 2018). Studies utilizing *in vitro* perfused hearts have reported that enhancement in workload is enough to increase cardiac carbohydrate and fatty acid catabolism (Goodwin et al., 1998; Goodwin and Taegtmeyer, 2000; Zhou et al., 2008). Additionally, Riehle et al. (2014) have suggested that 5 weeks of swim training significantly increases glycolysis, glucose oxidation, and fatty acid oxidation in isolated mice heart perfusions. Utilizing *in vivo* experiments, Bergman et al. (2009a, b) found that atrial pacing-induced the myocardium to utilize more fatty acids, glucose, and lactate to meet energy demand without affecting circulating substrates during increased heart workload. Epinephrine increases glucose catabolism partly through the activation of phosphofructokinase in hearts experiencing an elevated workload (Clark and Patten, 1981).

One more thing, oxidation of fatty acids is the main source of ATP production in the heart (Taegtmeyer et al., 2016a,b; Gibb and Hill, 2018). During exercise, catecholamine-activated lipolysis in adipose tissue elevates circulating free fatty acid (FFA) levels to 2.4 mmol/L. Fat oxidation then increases FFA uptake and utilization efficiency. The effects of exercise on fatty acid oxidation are inconsistent, including reports that it is increased (Burelle et al., 2004; Riehle et al., 2014), decreased (Hafstad et al., 2011), or unaffected (Gibb et al., 2017). After 10 weeks of HIIT, fatty acid oxidation decreased, while glucose oxidation increased in the myocardium of mice, which contributed to a maximal 12% decrease in myocardial oxygen consumption (Hafstad et al., 2011). The dissipation of myocardial oxygen is primarily due to increased mitochondrial uncoupling, induced by either fatty acid oxidation (Himms-Hagen and Harper, 2001) or ROS (Echtay et al., 2002).

Interestingly, the heart can also easily consume excess circulating lactate (up to 10 mmol/L) that is produced by muscular glycolytic activity during intense exercise (Lassers et al., 1972; Brooks, 2009). Lactate use is nearly 40% of ATP production during exercise (Schonekess, 1997). Even relatively low-intensity exercise can elevate lactate oxidation (Gertz et al., 1988), which may also promote fat oxidation to produce ATP under high workloads in the heart (Goodwin and Taegtmeyer, 2000).

Likewise, the specific type of strenuous exercise (like resistance exercise and long-term endurance exercise) lowers blood glucose levels (Coyle, 2000), while high-intensity aerobic exercise may increase blood glucose levels (Kemppainen et al., 2002). Similarly, myocardial glucose uptake and oxidation display the same trend as detected in human studies during exercise (Gertz et al., 1988; Kemppainen et al., 2002). These findings revealed that exercise displayed diverse effects on circulating glucose levels, and myocardial glucose utilization relied on the type, intensity, and duration of exercise. Epinephrine increases glucose catabolism partly through the activation of phosphofructokinase (Clark and Patten, 1981). Gibb et al. (2017) indicated that 4 weeks of treadmill running decreased glucose utilization via glycolysis during exercise and the early recovery period after exercise in mice. However, upon adaptation and full recovery, steady-state glycolysis rates appear to be increased in the heart (Gibb et al., 2017). This study also indicated that kinase-deficient 6-phosphofructokinase/fructose-2,6-bisphosphatase transgenic (Glyco^*L*^°) mice appear to regulate genes sufficiently (e.g., C/EBPβ and CITED4) (Bostrom et al., 2010; Bezzerides et al., 2016) partially related to exercise-induced cardiac growth (Gibb et al., 2017). The above results show that alterations in glycolysis caused by exercise are essential regulators of the cardiac growth process.

Furthermore, the adaptation to exercise is also regulated by hormones, like insulin-like growth factor 1 (IGF-1) (Kim et al., 2008) and neuregulin 1 (Waring et al., 2014; Cai et al., 2016), which are increased during exercise adaptation and likely promote glucose uptake and utilization. Metabolic alteration induced by these hormones is partially mediated by Akt (Pozuelo Rubio et al., 2003), which may increase glycolysis by activation of the myocardial form of 6-phosphofructokinase/fructose-2,6-bisphosphatase (PFK2) (Deprez et al., 1997; Mouton et al., 2010). Akt is an essential regulator of exercise-induced cardiomyocyte regeneration and plays a cardioprotective role through the up-regulation of the glucose transporter 4 (GLUT4) during ischemia-reperfusion (IR) injury (Wang et al., 2015).

Collectively, the metabolic adaptations that occur in the heart in response to exercise have yet to be elucidated. Research on the effects of exercise-induced myocardial energy metabolism primarily depends on data acquired from gene expression analysis or enzymatic activity assays. Moreover, discrepancies in different studies may be due to different exercise models, animal strains, or tissue acquisition protocols. There is still a lack of research on the relationship between the metabolic changes caused by exercise and the activation of adaptive programs in the regeneration of cardiomyocytes. Additionally, the importance of substrate metabolism, intermediates, or final products for exercise-induced heart regeneration remains unclear. Therefore, careful design of exercise training plans is required.

### Exercise Modulates Oxidative Stress in Cardiomyocytes

The mechanisms of exercise-induced oxygen reduction and oxygen metabolism alteration also include increased myocardial antioxidant capacity, decreased ROS accumulation, and thus, diminished ROS-induced mitochondrial uncoupling (Starnes et al., 2007; Bo et al., 2008). Starnes et al. (2007) found that ROS production decreased in rat myocardial mitochondria after 16 weeks of treadmill running. Bo et al. (2008) found that 6 weeks treadmill running could promote mitochondrial efficiency of oxidative phosphorylation through a decrease in ROS production and an increase in ROS elimination in rat hearts. Both short-term (10 days of treadmill training) (Hyatt et al., 2016) and long-term regular exercise (Radak et al., 2013; Ghiasi et al., 2015) can increase antioxidant enzymes and enhance oxidative stress resistance in the heart. Even 6 weeks of voluntary running with uneven exercise intensity can reduce oxidative stress in diabetic rats (Naderi et al., 2015; Chodari et al., 2019).

Further, decreases in oxidative stress and ROS production have also been detected in mice with diabetic cardiomyopathy following 16 weeks of running exercise (Wang et al., 2020). Moreover, 8 weeks of resistance exercise (Effting et al., 2019) or swim training (de Farias et al., 2013) can modulate redox imbalance and reduce oxidative damage in the myocardium in mice with diet-induced obesity. Exercise elevates the protein expression of several mitochondrial antioxidant enzymes, including superoxide dismutase 1 (SOD1) and superoxide dismutase 2 (SOD2), as well as the H_2_O_2_ removing enzymes glutathione peroxidase-1 and catalase. Thus, the exercise-induced reinforcement of antioxidant enzymes has a positive effect on the promotion of ROS elimination in myocardial mitochondria (Judge et al., 2005). However, whether the enhancement of ROS elimination and increased antioxidant levels induced by exercise in the myocardium can facilitate a reduction of oxidative DNA damage and activation of cardiomyocyte regeneration require further confirmation.

Numerous studies reveal that exercise training protects cardiac function through induced alterations in mitochondrial phenotype and biogenesis (Lee et al., 2012; Powers et al., 2014; Tao et al., 2015; Trewin et al., 2018; Boulghobra et al., 2020), which is beneficial to exercise-induced cardiomyocyte regeneration. Exercise-associated changes in the redox milieu regulate several factors of mitochondrial biogenesis, such as adenosine monophosphate-activated protein kinase (AMPK), Sirtuins 1 (Sirt1), peroxisome proliferator-activated receptor-γ coactivator 1α (PGC-1α), and mitogen-activated protein kinase (MAPK). In hearts undergoing exercise training, ATP is consumed rapidly to upregulate the AMP/ATP ratio and increase the activity of AMPK, which induces a series of phosphorylation-dependent modification cascades of factors like PGC-1α (Hardie et al., 2006). The activity of cardiac AMPK increases progressively with exercise intensity during treadmill running (Coven et al., 2003). Additionally, AMPK promotes translocation of GLUT4 to the cardiomyocyte membrane, which induces the upregulation of PFK2 and downstream fructose 2,6-bisphosphate to increase glycolysis (Marsin et al., 2000). After swim training, decreases in ROS production and an increase in antioxidative enzyme expression can be detected through the activation of AMPK in rat hearts (Ma et al., 2015; Wang et al., 2016). Sirt1 is a NAD^+^-dependent enzyme that regulates cardiomyocyte energy and functions through enhancing deacetylase activity (Bugger et al., 2016). Since Sirt1 relies on the NAD^+^/NADH ratio, it is sensitive to alterations induced by exercise in cardiomyocytes, such as cellular energy metabolism and redox status. The overexpression of Sirt1 can promote cardiomyocyte proliferation, with cardiac regeneration being demonstrated in both *in vivo* and *in vitro* studies (Li B. et al., 2018; Li et al., 2019). HIIT has been shown to upregulate the Sirt1 mRNA expression in diabetic rats and has been found to be more effective than continuous exercise training (Khakdan et al., 2018). Long-term running dramatically increases Sirt1 and PGC-1α protein expression in the rat heart (Bayod et al., 2012). PGC-1α is essential for mitochondrial density increment to ameliorate the efficiency of ATP production by reducing mitochondrial respiration in cardiomyocyte after exercise training (Watson et al., 2007; Wang et al., 2020). The concentration of PGC-1α is upregulated in the myocardium of mice by swimming (Sylviana et al., 2018) and running (Li et al., 2011) exercises. The promotion of AMPK/PGC-1α signal transduction induced by treadmill running is related to decreased ROS accumulation in the rat myocardium (Wang et al., 2020). The interconnecting role of AMPK-Sirt1 and PGC-1α plays a regulatory role in cardiomyocyte mitochondria metabolism. For instance, 4 weeks of running training has been shown to promote the activity of the Sirt1/PGC-1α/phosphatidylinositol three phosphate kinase (PI3K)/Akt signaling pathway in MI rat cardiomyocytes (Jia et al., 2019). Moreover, exercise can improve mitochondrial biogenesis, prevent diabetic cardiomyopathy-associated inhibition of PGC-1α, and activate Akt signaling in mice with diabetic cardiomyopathy (Wang et al., 2015).

Although the positive role of exercise in regulating decreases in ROS accumulation, increases in antioxidative capacity, and promotion of mitochondrial biogenesis in cardiomyocytes is definite, possible molecular mechanisms remain controversial. However, clarifying the specific relationship between modulation of exercise in oxidative stress and regeneration in cardiomyocytes is complicated, due to the variety of exercise models and physiological conditions.

## Oxygen Metabolism Related Molecular Changes in Exercise-Induced Cardiomyocyte Regeneration

**FIGURE 3:**
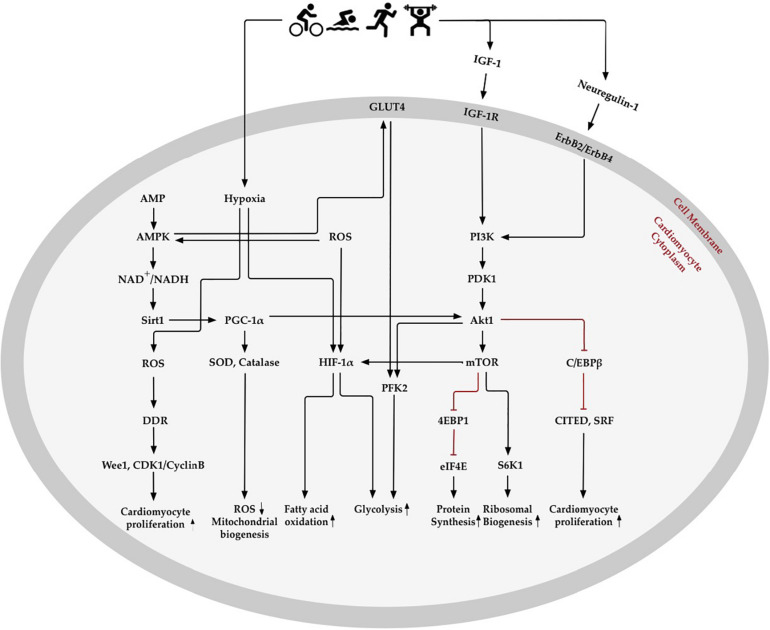
Oxygen metabolism-related pathways in exercise-induced cardiomyocyte regeneration. Exercise stimulates physiological signaling pathways, such as those involved in creating mild hypoxia, changes in fatty acid and glycolysis metabolism, elimination of reactive oxygen species (ROS), enhancement of antioxidative capacity, and regulation of the oxygen metabolic molecular pathway in the heart. AMP- activated protein kinase (AMPK) enhances mitochondrial biogenesis and energy metabolism through Sirtuins1 (Sirt1) and peroxisome proliferator-activated receptor-γ coactivator 1α (PGC-1α), which increase antioxidative enzymes to promote ROS elimination and mitochondrial biogenesis. Exercise-induced hypoxia and increase in ROS content promotes hypoxia-inducible factors 1α (HIF-1α) expression, which decreases fatty acid oxidation and increases glycolysis. Insulin-like growth factor 1 (IGF-1) and neuregulin-1 activate phosphoinositide 3-kinase (PI3K) and downstream Akt signaling pathways. Akt activates 4EBP1 and S6K1, downstream of the mechanistic target of rapamycin (mTOR), which may promote protein and ribosomal synthesis. *Akt, RAC-*α *serine/threonine-protein kinase; CDK1, cyclin dependent kinase 1; C/EBP*β, *CCAAT/enhancer binding protein-*β*; CITED4, CBP/p300-interacting transactivator 4; DDR, DNA damage response; eIF4E, translation initiation factor eIF4E; GLUT4, glucose transporter 4; IGF-1R, IGF-1receptor; PDK1, phosphoinositide-dependent protein kinase-1; PFK2, 6 phosphofructokinase/fructose-2,6-bisphosphatase; S6K1, ribosomal protein S6 kinase-*β*1; SOD, superoxide dismutase; SRF, serum response factor.*

The changes of oxygen metabolism might activate and interact with extracellular and intracellular signaling pathways to promote cardiomyocyte regeneration (Figure 3) (Bo et al., 2020). Exercise-induced cardiac growth is mediated mostly through increased insulin growth factor-1 (IGF-1) signaling in the hearts of athletes (Serneri et al., 1999). The beneficial paracrine effect of IGF-1 in the heart acts via a downstream signaling pathway by reducing ROS generation and oxidative DNA damage (Kajstura et al., 2001; Yang et al., 2005). Results from transgenic mice models show that the IGF-1-PI3K-Akt1 axis is critical to mediate exercise-induced cardiomyocyte regeneration (DeBosch et al., 2006; Medeiros et al., 2011; Ma et al., 2013; Palabiyik et al., 2019). For example, improved physiological growth of the heart can be induced by overexpression of the PI3K (p110α) in mice (McMullen et al., 2004), while the physiological hypertrophy of heart is unable to detected in dominant-negative PI3K (p110α) transgenic mice (McMullen et al., 2003). Akt1 is a serine/threonine-protein kinase to regulate cell cycle through extending the half-life of cyclin D (Jia et al., 2008; Parekh et al., 2011). The activation of Akt1 and downstream effectors of mTOR, including S6K 1 and 4EBP1, are considered to be essential for modulating cardiac growth by regulating protein biosynthesis (Shiojima and Walsh, 2006). Moreover, swimming exercise increased the activity of the PIK3/Akt1/mTOR signaling pathway in rats after 8 weeks of training (Ma et al., 2013; Palabiyik et al., 2019). Endurance exercise reduced C/EBPβ expression, which was activated by upstream of Akt1 and PGC-1. The downregulation of C/EBPβ promoted cardiomyocyte proliferation through negative regulation of CITED4 (Bostrom et al., 2010).

The neuregulin-1/ErbB2/ErbB4 pathway is another critical signaling pathway that changes in response to physical exercise and can stimulate the intracellular Akt signaling pathway by binding to the receptor on the cardiomyocyte membrane. The specific function of neuregulin-1 is to induce differentiated cardiomyocytes to reenter the cell cycle from S phase and experience both karyokinesis and cytokinesis, leading to cardiomyocyte proliferation in adult cardiomyocytes (D’Uva et al., 2015). Both low (55–60% of individual VO_2__*max*_) or high (85–90% of individual VO_2__*max*_) intensity of running can increase the number of newly formed cardiomyocytes through upregulated neuregulin-1 expression on rat hearts (Waring et al., 2014). It has been further demonstrated that 5 weeks of running exercise can increase neuregulin-1 expression and stimulate the downstream ErbB2, ErbB4, and PI3K/Akt signaling pathways to activate endogenous cardiac regeneration in MI rats (Cai et al., 2016).

## Can Exercise Induce Cardiac Regeneration Through Oxygen Metabolism in Humans?

Notably, alteration of oxygen environment and oxygen metabolism in the myocardium can trigger cardiomyocyte regeneration in the adult heart, especially when the environment becomes hypoxic (Puente et al., 2014; Nakada et al., 2017). Despite the lack of evidence that hypoxia induces cardiac regeneration in humans, hypoxic treatment may provide an entirely new therapeutic direction. For example, 3 weeks of passive intermittent short-term (3–5 min) hypoxia exposure (fraction of oxygen 10–21%) increased aerobic capacity and exercise tolerance in older men with and without coronary artery disease (Burtscher et al., 2004). Hypoxic therapy (inhaled oxygen concentration fraction 10–21%) will also lead to lower surgical risks compared with tissue regeneration *in vitro* and putative cardiac transplantation. Data suggests that the oxygen concentration that can activate cardiomyocyte regeneration is extremely low (7% air-saturation) (Kimura et al., 2017). This degree of hypoxia is equivalent to the oxygen concentration at the summit of Mount Everest (8,848 m). However, before the administration of hypoxic therapy, the “skean” effect of extreme hypoxia, which might bring fatal complications to organs like the brain and kidneys, should be considered. Under the regulation of neurohumoral factors, exercise can redistribute blood and oxygen throughout the body. As elucidated above, a mildly hypoxic environment and the reduction of oxidative damage induced by exercise in the myocardium can safely activate and promote cardiomyocyte regeneration.

## Summary and Future Perspective

Collectively, exercise, as an intensity stimulation, can alter the oxygen environment and oxygen metabolism in the myocardium, including creating a mildly hypoxic environment, changing energy metabolism, promoting ROS elimination, enhancing antioxidative capacity, and regulating oxygen metabolic molecular pathway in the heart. According to the studies discussed above, these exercise-induced alterations have a significant positive effect on the activation and promotion of cardiomyocyte regeneration. A full determination of the regulatory role of oxygen metabolism and related factors during and after exercise in cardiomyocyte regeneration will provide biological insight into endogenous cardiac repair mechanisms.

However, the potential molecular mechanisms are not so much clear regarding the interactions between environmental oxygen-dependent metabolic switch in cardiomyocytes and exercise training. The future studies in the oxygen metabolism of exercise-induced cardiomyocyte regeneration require systematic and specific exercise models in extensive animal studies to clarify the molecular pathway, and to evaluate the efficacy of exercise. In particular, upstream signaling pathways, transcription factors, epigenetic modifiers, and mitochondrial function of cardiomyocyte involved in regulating the metabolic shift from a non-proliferative state to a regenerative state in the context of exercise, are essential research directions. Moreover, the effects of exercise depend on the type (aerobic/resistance), intensity (mild/moderate/intense/exhaustive), frequency (sessions per day/week/month), and subject characteristics (age, sex, endurance capacity, and health condition). Further work needs to address all these issues, providing a foundation for exercise as a cost-effective intervention to promote cardiomyocyte regeneration and restore cardiac function in clinical treatment.

## Author Contributions

BB conceived of the study, collected the data and material, and wrote the manuscript. SL helped collect and analyze the data and draft the manuscript. KZ helped draft the manuscript. JW helped conceive of the study, and revise the manuscript. All authors gave final approval for publication.

## Conflict of Interest

The authors declare that the research was conducted in the absence of any commercial or financial relationships that could be construed as a potential conflict of interest.
